# Comparing Different Epinephrine Concentrations for Spinal Anesthesia in Cesarean Section: A Double-Blind Randomized Clinical Trial

**Published:** 2015-07

**Authors:** Arash Hamzei, Seyed Hossein Nazemi, Ali Alami, Arezoo Davarinia Motlagh Gochan, Azizollah Kazemi

**Affiliations:** 1 Department of Anesthesiology and Operation Room, Gonabad University of Medical Sciences, Gonabad, Iran;; 2 Social Determinants of Health Research Center, Department of Public Health, School of Public Health, Gonabad University of Medical Sciences, Gonabad, Iran;; 3 Department of Critical Care Nursing, Gonabad University of Medical Sciences, Gonabad, Iran;; 4 Department of Anesthesiology, 22-Bahman Hospital, Gonabad University of Medical Sciences, Gonabad, Iran

**Keywords:** Cesarean section, Spinal anesthesia, Paralysis, Analgesic, Hemodynamics

## Abstract

**Background:**

Although various anesthetic techniques can be used in different kinds of surgeries, spinal anesthesia has received considerable attention for the lower abdomen and lower extremities surgeries and cesarean section. This study aimed at comparing the effect of adding epinephrine 1:1000 and 1:10000 to lidocaine and fentanyl in spinal anesthesia on the prolongation of paralysis, analgesia and hemodynamic changes in pregnant women candidate for cesarean section.

**Methods:**

A double blind randomized clinical trial was carried out on 66 pregnant women (equally sized control and treatment groups of 33) in 2011. After randomizing the participants into two groups of recipients of epinephrine 1:1000 plus lidocaine 5% and fentanyl (control group) and recipients of epinephrine 1:10000 with lidocaine 5% and fentanyl, (treatment group), the participants’ systolic and diastolic blood pressure and heart rate were recorded before and 1, 3, 5, 10, 15 minutes after procedure. Besides the prolongation of paralysis and analgesia, the presence of postoperative nausea and vomiting were evaluated. The outcome of the study was analyzed using SPSS software and via *t* test, χ2 test and RMANOVA.

**Results:**

The mean age (standard deviation) of the participants was 29.3 (4.4) and 28.2 (4.5) in the treatment and control groups, respectively. There were no statistical significance between the participants’ prolongation of paralysis, analgesia, the frequency of nausea and vomiting, and the average values of hemodynamic variables between the two groups.

**Conclusion:**

The use of epinephrine 1:10000 along with lidocaine and fentanyl is recommended in spinal anesthesia in pregnant women candidate for cesarean section.

**Trial Registration Number: **IRCT201012225445N1.

## Introduction

Although various types of anesthetic techniques are currently used in different operations, considerable attention has been paid to spinal anesthesia.^1^ It is a form of regional anesthesia involving the injection of a local anesthesia into the subarachnoid space, in the surgery of the lower abdomen, lower extremities, and Cesarean Section (CS).^1^ In spinal anesthesia, epinephrine is used to prolong paralysis and anesthesia obtained by local anesthetics such as bupivacaine, procaine, tetracaine, and lidocaine.^1^ Spinal anesthesia is commonly obtained using 5% lidocaine within 7.5% dextrose^2^ and fentanyl also used as an adjuvant to produce anesthesia and analgesia in different ways such as spinal block.^3^

Anesthesiologists often prepare epinephrine-containing local anesthetic solutions for regional anesthesia.^4^ Due to its vasoconstrictive effects; epinephrine has multiple benefits such as increasing the block length, producing optimal decrease in plasma anesthetic concentration and consequently decreasing adverse drug reactions.^5^ The mechanism of action of epinephrine has been explained in many studies.^6^ For instance, it has been proven that the addition of epinephrine to lidocaine can improve the quality of the block, prolong paralysis and analgesia and produce more favorable recovery outcomes in patients.^7^ It was demonstrated that, epinephrine increases the length of block through a reduction in local blood flow.^8^ The hemodynamic changes in the mother during spinal anesthesia were examined in various studies.^9-11^

CS is commonly performed in the case of previous history, complex delivery, fetal distress and/or breech presentation.^12^^,^^13^ There has been a rapid increase in the number of CS performed in recent years around the world,^14^^,^^15^ which may also be related to an increase in the number of women requesting CS or having a history of CS.^16^ There are many evidences, which indicate a dramatic increase of regional anesthesia application in CS.^17^^,^^18^

Up until recently, the only available drug form of epinephrine for spinal anesthesia implementation was ampoules of epinephrine 1:1000 injected at a dose of 0.2 ml accompanied with other drugs. But, a new medicinal form of epinephrine 1:10000 in ampoules exists in Iran. One of the important questions propounded is whether different concentrations of epinephrine cause different complications or different remedial effects. Inasmuch as no research has ever been carried out on the comparison of the effects of the two different forms of epinephrine in spinal injection, this research was done with the aim of comparing the effects of epinephrine 1:1000 and 1:10000 with lidocaine and fentanyl on the prolongation of paralysis, analgesia and hemodynamic changes in spinal anesthesia for CS. 

## Patients and Methods

This was a double blind, parallel group, randomized clinical trial study conducted in the city of Gonabad, in 2011. The patients composed of all pregnant women referring to the operating room at the 22-Bahman Hospital of Gonabad undergoing CS at the time of the study. According to previous researches,^19^ we selected 66 women that were candidate for pregnancy termination by cesarean via Feinstein cut. They were aged between 20 to 35 years; having no history of diseases consisting of diabetes, high blood pressure or clotting disorders and having no prohibition for the consumption of epinephrine. The exclusion criteria for the participants included the failure of spinal anesthesia for any reason, not having spinal anesthesia level between T4 and T6 and a change in the type of incision during CS for any reason. We entered all qualified mothers in the study during 6 months (from January to July, 2011) after receiving the participants’ verbal consent. According to a study by Yaraghi et al.^20^ the sample size was determined to be 30 participants in each group, but in order to avoid probable exclusion problems, 33 pregnant women were allocated to each group (i.e. 10% more).

$$N=\left[\frac{{2(Z_{1-\alpha /2}+Z_{1/\beta })}^{2}}{{∆}^{2}}\right]=\left[\frac{{2(1.96+0.84)}^{2}}{0.52^{2}}\right]=30$$

$$∆=\left(\frac{\left(\mu_{1-}\mu_{2}\right)}{\delta }\right)$$

α=0.05

β=0.20

After recording the exact information, the participants were allocated into study groups using balanced block randomization with a block size of 4. There were two study groups consisting of the control (recipients of lidocaine/fentanyl/5% and epinephrine 1:1000) and treatment groups (recipients of lidocaine/fentanyl/5% and epinephrine 1:10000). The prescribed drug for each participant composed of lidocaine 75 mg, fentanyl 25 micg and epinephrine 0.2 cc. For randomization procedures, initially the form of epinephrine prescribed was written on 66 cards and then each card was placed into a bag marked with a non-repetitive 3-digit code. Then the exact specifications of the patients were recorded and the related parts of the questionnaire were filled out by the main researcher followed by preparing the mother for CS in the operation room. Then, after a ready signal was received from the person injecting the anesthetic drug, the first researcher selected the corresponding code and drew up the specified amount of epinephrine into the syringe and was delivered to the administrator to be used. This procedure was repeated for all participants. The data gathering tool consisted of a questionnaire made by the researcher, including participants’ demographic characteristics, quality of paralysis, analgesia and hemodynamic changes consisting of Systolic Blood Pressure (SBP), Diastolic Blood Pressure (DBP), and Heart Rate (HR). The quality of the paralysis was evaluated from one hour after the initiation of spinal anesthesia and repeated every 15 minutes. The duration from the initiation of spinal anesthesia to the observation of the first dorsiflection of the participants’ foot was considered as the length of paralysis for each sample. Hemodynamic indices of the participants were measured before and in 1, 3, 5, 10, and 15 minutes after the spinal anesthesia. In this study, the first request of the participants for a pain reliever was considered as a measure indicating the end of analgesia and thus the time interval between the spinal injection and their requests is considered as the length of analgesia. It should be noted that neither the mothers nor the persons injecting the drugs as well as the observers participating in the study were aware of the concentrations of the epinephrine used. As a result, the research was double blind. 

Gathering the data, we used a questionnaire which was validated via face and content validity and its reliability was confirmed using the Cronbach’s alpha coefficient (alpha=0.86). The gathered data were entered in the SPSS software (version 16) and were analyzed using *t* test (the length of paralysis and analgesia), χ2 test (post-operative nausea state), and repeated measure ANOVA (SBP, DBP, HR). In this research, P<0.05 was considered as statistically significant.

Before starting the study, we received the approval from the Ethics Committee of Gonabad University of Medical Sciences. During the research process, written consent forms were received from the participants. The participants were also given the right to withdraw from the study at any stage of the research at free will. 

## Results

**Figure 1:**
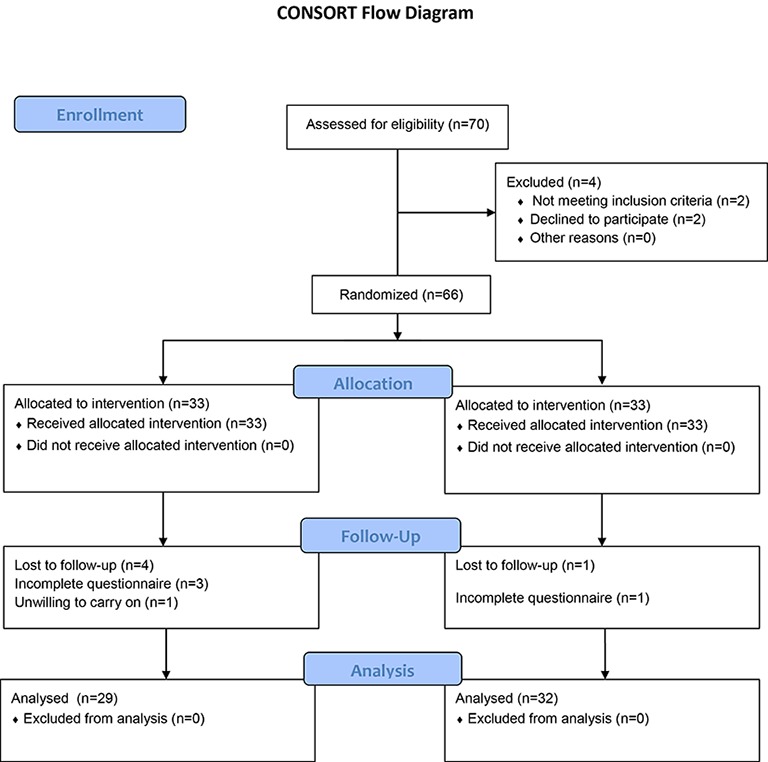
Shows the participant flow of the participants during our study.

In this study, there were 66 participants (33 in each of the two groups) who referred to the operation room of Gonabad’s 22-Bahman Hospital for CS from January to June 2011. The trial continued till the required participants were selected. Figure 1 shows the flowchart of participants during the study.

As can be seen, four participants in the treatment group and one pregnant woman in the control group were excluded. Consequently, 61 questionnaires (29 in the treatment and 32 in the control groups) were analyzed. Table 1 shows the mean (SD) of the study variables before treatment in both groups. The participants’ age range was 15 in both the treatment and control groups.

**Table 1:** The comparison of age, blood pressure, heart rate and previous cesarean section before intervention

**Variable names**	**Group**	
	**Treatment (n=29)**	**Control (n=32)**
Age (year), Mean±SD	29.3±4.4	28.2±4.5
SBP (mmHg), Mean±SD	115.9±13. 5	119.1±17.5
DBP (mmHg), Mean±SD	73.6±10.3	77.5±9.8
HR, Mean±SD	94.2±11.4	97.0±15.0
Previous cesarean section number (%)	18 (62.1%) 11 (37.9%)	17 (54.8%) 14 (45.2%)

**Figure 2:**
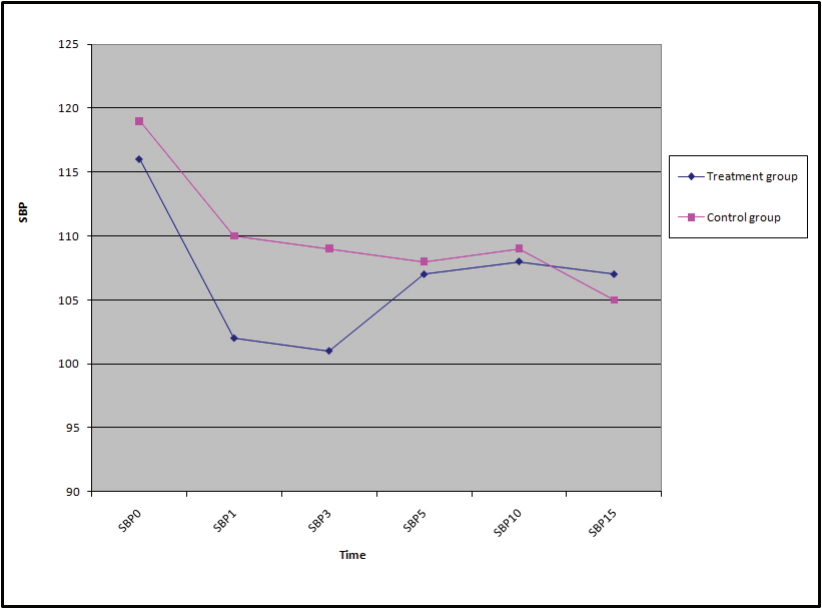
Trend of Mean Systolic Blood Pressure between treatment and control groups.

**Figure 3:**
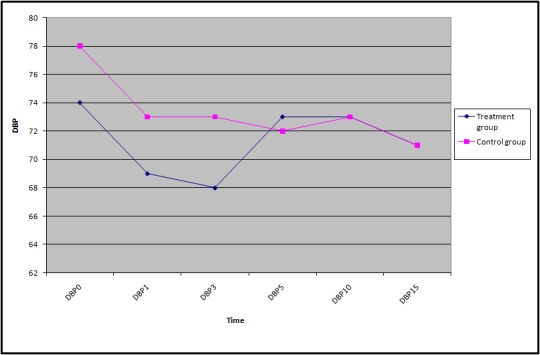
Trend of Mean Diastolic Blood Pressure between treatment and control groups.

**Figure 4:**
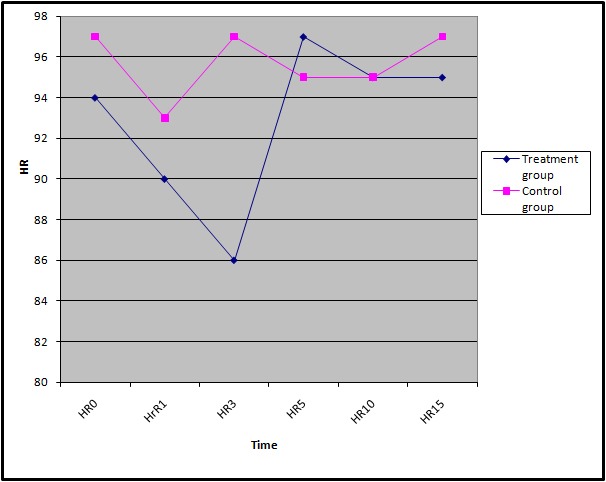
Trend of Mean Heart Rate between treatment and controul groups.

As can be seen in table 1, there was no considerable difference between the investigated variables between the treatment and control groups. Figures 2 to 4 shows the trends of SBP, DBP, and HR of the participants during the study.

The means (SD) of the participants’ SBP, DBP, and HR are shown in table 2. According to the findings, except for the mean heart rate, which was taken 3 minutes after the spinal anesthesia, there were no statistically significant differences between all the reported means in the treatment and control groups.

**Table 2:** Comparing the effect of epinephrine 1:1000 (control) and 1:10000 (treatment) on the heart rate, systolic and diastolic blood pressure

**Variables name**	**Time after intervention (minute)**	**Group**	
		**Treatment**	**Control**
Mean±SD systolic blood pressure	1^st^	102.07±19.53	110.31±20.87
	3^rd^	101.03±16.11	108.75±20.91
	5^th^	106.55±14.71	107.97±17.03
	10^th^	107.93±14.30	109.38±10.76
	15^th^	106.72±11.67	105.31±9.50
Mean±SD diastolic blood pressure	1^st^	68.62±9.53	72.50±13.44
	3^rd^	67.76±10.32	72.66±12.18
	5^th^	72.76±9.96	71.88±11.13
	10^th^	73.28±7.82	73.12±7.80
	15^th^	71.38±10.26	71.41±7.10
Mean±SD heart rate	1^st^	89.76±16.20	93.34±18.80
	3^rd^	85.83±13.28	97.09±21.80
	5^th^	97.07±17.67	94.59±17.18
	10^th^	94.90±16.91	94.53±18.02
	15^th^	94.66±15.44	97.03±17.71

Tables 3 and 4 show comparative results between the treatment and control groups after the intervention. As can be seen, there were no significant differences between the participants’ SBP, DBP, HR, duration of paralysis and analgesia, and the frequency of post-operative nausea and vomiting of the two groups.

**Table 3:** Comparative results between the treatment and control groups after intervention in terms of hemodynamic variables

**Variable**	**Between subject**	
	**F**	**P value**
Systolic blood pressure	1.586	0.213
Diastolic blood pressure	1.169	0.284
Heart rate	0.854	0.359

**Table 4:** Comparative results between the treatment and control groups after intervention in terms of the length of paralysis, analgesia and frequency of post-operative nausea and vomiting

**Variable name**	**Group**		**P value**
	**Treatment (n=29)**	**Control (n=32)**	
Mean±SD analgesia length (min)	162.8±51.6	179.4±48.7	0.679
Mean±SD paralysis length (min)	116.9±26.5	127.9±27.6	0.340
Postoperative nausea and vomiting			
Yes number (%)	11 (37.9%)	8 (25.8%)	0.313
No number (%)	18 (62.1%)	23 (74.2%)	

There was no protocol deviation in this study

## Discussion

Spinal anesthesia for surgery on the lower abdomen, lower extremities and CS has been the focus of considerable attention. In this technique, a local anesthetic is injected into the subarachnoid space and results in regional paralysis and analgesia. To prolong the duration of analgesia, a narcotic drug is used together with the local anesthetic.^1^ This study aimed at comparing the effects of epinephrine 1:1000 and 1:10000 on patients. The result of this research is considered to be an appropriate reference on the subject of using different concentrations of epinephrine.

Our study ascertained that using epinephrine 1:10000 instead of 1:1000 along with lidocaine and fentanyl produces no different effects on the duration of paralysis and analgesia, nausea and vomiting, SBP, DBP, and HR.

Numerous studies have been conducted on investigating and comparing the drug-combined effects on the length of paralysis. Kito et al.^5^ reported higher quality and longer duration of block in using epinephrine with local anesthetic when used in spinal anesthesia. But no other effects on the level and length of analgesia was reported when using different doses of epinephrine. The results of the above mentioned research are not exactly comparable to our findings as they include the comparison of different doses of epinephrine whereas we have compared different concentrations of epinephrine. It is noteworthy that there was no statistically significant difference in the length of the pain relief when either the dose or concentration of the epinephrine was changed.^5^

Santos et al.^6^ reported the results of their study comparing the effect of epinephrine 1:100000 and 1:200000 on soft tissue anesthesia in candidates for molar tooth extraction. They stated similar postoperative pain relief and insignificant and comparable changes in hemodynamic in the two groups. Additionally, they recommended that the use of lower concentrations of epinephrine is effective.

Gurbet et al.^19^ studied the effect of adding epinephrine to bupivacaine/fentanyl mixture routinely used in interathecal injection in spinal anesthesia during delivery. They indicated that an addition of lower dose of epinephrine (25 µg) produces the best effect. The results of our study are almost in line with the results of the latter two studies. One difference might be a lower percentage of those pregnant women who suffered from nausea and vomiting in Gurbet et al. study compared with our research. This would be linked to the method of terminating pregnancy and the method of evaluating pain in pregnant women.

Muthukumar et al.^21^ compared the hemodynamic responses following the addition of different concentrations of epinephrine to lidocaine for surgical field infiltration during cleft palate surgery in children. They discovered that increasing the concentration of epinephrine from 1:400000 to 1:200000 led to the occurrence of tachycardia in the samples. This result does not correspond with the outcomes of the present study and may be associated with the difference in the form of epinephrine prescribed or even to the age of the samples.

Random allocation of the participants to the treatment and control groups as well as concealment of this allocation is considered as the strengths of the present study. We can nevertheless confirm that there were some limitations in our research. The pregnant women participating in this study were chosen from one center; therefore this research is a one-center study. Although the hospital from which the samples were selected is the only hospital in Gonabad, the possibility of selection bias cannot be ruled out. The method used for evaluating the length of analgesia could be another weakness. With regards to the different levels of pain threshold in people and due to inter personal variation, the results of this part of the study could be uncertain. 

The present study can be used by anesthetic specialists, operation room personnel and hospital heads. There were not only no significant differences in the remedial effects of using lower concentrations of epinephrine in spinal anesthesia compared with the higher concentrations, but it may also cause a decrease in complications resulting from drugs with higher concentration entering the circulation. We recommend performing similar studies with a longer term and with larger volumes of sample in multiple centers. 

## Conclusion

The use of epinephrine 1:1000 or 1:10000 along with lidocaine and fentanyl in spinal anesthesia in women candidate for CS produced no statistically significant difference in the hemodynamic, post-operative nausea and vomiting, and post-operative paralysis and pain relief. Hence, the use of lower concentrations of epinephrine is recommended. In other words, it would be practical to replace the commonly used form of epinephrine 1:1000 with epinephrine 1:10000 at the pharmaceutical market level so that the new packaging could be found more conveniently for spinal anesthetic practices. 
